# Vermont State Dental Society

**Published:** 1892-08

**Authors:** 


### PROCEEDINGS.

### Vermont State Dental Society.

The sixteenth annual meeting of the Vermont State Dental Society held at the Van Ness House, Burlington, on the sixteenth, seventeenth and eighteenth of March, was one of the best attended and most interesting of any held thus far, fifty-five being present at the first session, the number being increased to eighty-five the next morning. Among them were several prominent dentists from other States and Canada.

The meeting was called to order at 7:30 o'clock Wednesday evening, and after the preliminary business had been disposed of, Dr. James Lewis, gave an address of welcome which was followed by papers from Dr. S. Hubbell, of Burlington, on "Immediate Root Filling;" Dr. G. W. Hoffman, of White River Junction, on "Dental Legislation," and Dr. C. W. Staples, Lyndonville, "A Practical Cheoplastic Plate," with models.

At the Thursday morning session, papers were read by the President, Dr. W. S. Curtis; Prof. J. H. Linsley, of New York, "Micro-organisms of the Mouth," demonstrating the action of these microbes upon different substances. All pronounced this paper one of the best ever read before the society. Dr. H. L. Cleaves, of Montpelier, wrote of "The Formation and Care of the Teeth," and Dr. Forest G. Eddy, of Providence, R. I., "Use of Gutta-Percha as a Root-Canal Filling." This paper showed large experience with this class of fillings. Dr. Eddy uses the crude gutta-percha dissolved in chloroform.

Thursday afternoon until four o'clock was devoted to clinics by the following gentlemen: Dr. C. A. Timme, of New York, demonstrated the making and setting of Enamel Inlays. The inlays being fused over the flame of an alcohol lamp.

Dr. F. S. Belyea, of Boston, Crown and Bridge-Work, making and setting three different bridges during the three days' meeting, which were very beautiful pieces of work, and proved Dr. Belyea a master of this class of dentistry.

Dr. G. O. Webster, of St. Albans, Staining Artificial Teeth by painting them with paints sold by Ash & Co., then burning them in a furnace. This work seems very nice when one wishes gold on artificial teeth or when representing a tobacco-chewer's teeth.

Dr. J. E. Waitt, Boston, exhibited the Packard Ether Inhaler with improvements by himself; a very ingenious apparatus for rapid anæsthesia with either ether or chloroform.

At 4:30 o'clock the meeting was again called to order, and a paper read by Dr. G. F. Cheney, "Pulp Protection by Cavity Lining," followed by an interesting paper by Dr. W. G. Beers, of Montreal, "Some Observations During Pregnancy." Dr. H. C. Merriam, Salem, Mass., followed on "Professional Journalism," and Dr. A. J. Parker, of Bellows Falls, on "First Dentition."

The entire evening was devoted to a banquet and social enjoyment.

At 9 o'clock Friday morning the meeting was called to order and officers for the ensuing year were chosen, as follows: Dr. Geo. F. Cheney, President; Dr. A. J. Parker, First Vice-President; Dr. W. H. Wright, Second Vice-President; Dr. Thos. Mound, Secretary; Dr. W. H. Munnsell, Treasurer. Executive Committee, Drs. E. O. Blanchard, Geo. O. Webster, and C. W. Staples. Dr. G. W. Hoffman, State Prosecutor.

After the newly elected president had taken the chair, papers were read by Dr. C. G. Campbell, St. Albans, "Pulpless Teeth," and Dr. W. H. Wright, "Dentists and Dentistry as seen by our Patients."

The meeting was adjourned to meet the third Wednesday in March, 1893, at St. Albans.

### Resolutions on the Death of Dr. W. H. Atkinson.

*To the Vermont State Dental Society, Gentlemen:*

It is recorded that Dr. William H. Atkinson, an honorary member of this society is dead. But *such men* never die. Their thoughts and deeds are immortal—they live forever.

His was a master mind, richly endowed with rare gifts. An original thinker, with a keen and swift insight into the heart of things.

He persistently opened his mind to nature and science and they rewarded him in a wonderful revelation of their secrets.

While he hated shams and a smattering of truth, he was generous toward all mankind. He had a heart that was as large as a mountain, and a love that was world wide. He gave all his powers of body, soul, and spirit, to the profession he loved so well, and to the friends whom he loved so much.

He was a grand man, a sincere man, a man of integrity, an honor to the dental profession.

He said many times before going hence: "Whenever you, my brethren, assemble in the interest of dental science, I will be with you in spirit."

We will ever welcome that spirit of love and progress, possessed in such large measure by our beloved brother.

We regret that we can no longer hear his words of wisdom, or feel the magnetic throb of his noble heart, but we bless and revere his memory.

J. L. PERKINS,
R. M. CHASE,
G. F. CHENEY,
*Committee.*

